# Automated Wormscan

**DOI:** 10.12688/f1000research.10767.3

**Published:** 2019-01-04

**Authors:** Timothy Puckering, Jake Thompson, Sushruth Sathyamurthy, Sinduja Sukumar, Tirosh Shapira, Paul Ebert

**Affiliations:** 1 School of Biological Sciences, University of Queensland, St Lucia, QLD, 4072, Australia; 2 Plant Biosecurity Cooperative Research Centre, Canberra, ACT, 2617, Australia

**Keywords:** WormScan, Caenorhabditis elegans, toxicology, software, phosphine

## Abstract

There has been a recent surge of interest in computer-aided rapid data acquisition to increase the potential throughput and reduce the labour costs of large scale *Caenorhabditis elegans* studies. We present Automated WormScan, a low-cost, high-throughput automated system using commercial photo scanners, which is extremely easy to implement and use, capable of scoring tens of thousands of organisms per hour with minimal operator input, and is scalable. The method does not rely on software training for image recognition, but uses the generation of difference images from sequential scans to identify moving objects. This approach results in robust identification of worms with little computational demand. We demonstrate the utility of the system by conducting toxicity, growth and fecundity assays, which demonstrate the consistency of our automated system, the quality of the data relative to manual scoring methods and congruity with previously published results.

## Introduction

Several techniques exist for digitisation and computational analysis of *Caenorhabditis elegans*, with the highest throughput techniques being those that utilise photo-grade flatbed scanners for image acquisition. The first of these, WormScan ^1^, lacks post-acquisition automation of data processing, whereas the second, The Lifespan Machine ^2^, is more complicated to implement and is computationally intensive. We present a very simple to implement, low-cost system based on automating elements of WormScan, which can score *C. elegans* on agar plates for mortality, size, and fecundity at a rate of tens of thousands of worms per hour with very little operator input.

*C. elegans* is the premier model organism for ageing and toxicological research. They are among the most intensively studied organisms of the last 50 years, which has resulted in the development of powerful genetic and molecular research tools. These tools include an extensive collection of readily available mutant strains ^3^, comprehensive and commercially available RNAi libraries ^4^, and a large collection of strains that each contain a GFP tagged gene. These strains provide multiple avenues by which genes of toxicological importance can be efficiently investigated, either individually or in combination. Additionally, the size, simplicity, and fecundity of the organism make it a popular choice for toxicological studies ^5– 7^. Furthermore, *C. elegans* have a very short natural lifespan, making them ideal for studies into the mechanisms of lifespan determination ^8– 11^. The ability to assay the worms in large numbers makes it possible to address the immense combinatorial challenge of testing large chemical libraries to identify bioactive compounds against a large numbers of strains.

There has been a recent surge of interest in rapid data acquisition to increase the potential throughput and reduce the labour costs of large scale *C. elegans* studies. In 2012, Mathew *et al*. developed WormScan, a low cost analysis tool that uses high resolution flatbed scanners and computer analysis to collect mortality data from *C. elegans* on petri dishes ^1^. This system replaces microscopy with commercially available transmission flatbed scanners, and replaces the mechanical stimulus of a platinum wire probe with the aversive stimulus of the bright light from the scanner, which was demonstrated to induce comparable responses. However, while WormScan employed computer image processing of scanned images to replicate human counting, the system was not automated. The 'Lifespan Machine', a more sophisticated, higher throughput system was then developed, which, like WormScan, relies on machine learning to identify worm-like objects ^2^. In its current implementation, this system requires sophisticated IT support.

Here we present an automated software system, Automated WormScan. It is extremely easy to implement and use. It employs low-cost hardware that can be set up by a person with average computer skills in under an hour, and can be used by any researcher with typical computer expertise. Any number of scanners can be run simultaneously from a single standard desktop PC. Three scanners permit an operator to count over ten thousand individual organisms per hour, with only approximately ten minutes of actual operator labour. By comparison, traditional microscope-based counting methods often count fewer than 500 individuals per hour of uninterrupted labour.

The automated system does not depend on machine learning for image recognition, but rather relies on an alternative worm counting method of WormScan, the generation of a difference image from sequential scans, with moving objects scored as live worms. This approach results in robust identification of worms with little computational demand. We demonstrate the utility of the system by conducting toxicity, growth and fecundity assays that demonstrate the consistency of our automated system, the quality of the data relative to manual scoring methods and congruity with previously published results. For this analysis, we use fumigant phosphine as a toxic stress as our lab has studied the toxicology of this compound quite extensively ^6, 7, 12– 16^. We do, however, routinely use the system to study the toxic or protective effects of a wide range of other gases and dissolved compounds as well.

## Methods

### Scanning

As with both WormScan and the recent Lifespan Machine system, an Epson v700 (or v800) photo scanner is used to take transmission scans of *C. elegans* grown on a thin lawn of *Escherichia coli* on agar growth medium in 6cm petri dishes ^1, 2^. In order to minimise the possibility that worms will be in physical contact at the time of scanning and accidentally counted as a single worm, up to 100 worms are scanned per plate. Scans are initially acquired as .tiff images in 16 bit greyscale at 2400 dpi, but are then saved as condensed jpeg images. This ensures sufficient pixel count to be able to distinguish small differences between sequential images, while minimising file size. Up to 12 populated petri dishes can be scanned at a time in each scanner, and all are scanned simultaneously. This is followed immediately by a second scan, each scan taking approximately ten minutes. While all experiments in this study used three scanners simultaneously, there is no absolute limit to the number of scanners that could be utilised in parallel.

### Image analysis and data concatenation

The software for automated analysis of the images exists as two components; a plugin for the FIJI image processing application ^17^, and a macro for Microsoft Excel. The FIJI plugin automates a sequence of image manipulations that generates a reliable count of the number of light-responsive individual organisms on each agar plate. The Excel macro then automates the extraction, labelling and concatenation of these counts into a spreadsheet.

### Worm strains and maintenance

Worm maintenance was carried out according to the standard procedures in Wormbook ^18^. Briefly, worms were grown on solid nematode growth medium seeded with *E. coli* OP50, and maintained at 20 degrees. Populations of nematodes were synchronised by dissolving gravid adult worms in 1.5% hypochlorite bleach and 0.75M NaOH to release their eggs. Eggs were immediately rinsed in M9 buffer and maintained overnight at room temperature in M9 buffer with gentle shaking to allow the eggs to hatch. The resulting L1 larvae were then transferred to NGM agar plates seeded with OP50 to initiate growth ^18^.

### Phosphine generation and treatment

Phosphine was generated as described in Cheng *et al.*, 2006 ^7^ by dissolving commercially available ammonium phosphate tablets ('Quickphos'; UPL ltd.) in 5% sulphuric acid in a glass Valmas Chamber. The resultant phosphine was then quantified using a phosphine gas monitor (Canary ‘SmarTox-O’ PLZZ) and injected through a septum into a sealed glass desiccator, to achieve the concentration required by the parameters of each experiment.

Worms were grown on NGM agar plates seeded with OP50 at 20°C for 48 hours, by which time they had reached the early L4 stage of development. Prior to exposure to phosphine, the number of live worms on each plate was determined by the Automated Wormscan procedure described in this paper. These plates were then sealed inside the desiccators prior to phosphine injection and were exposed to phosphine for 24 hours. Worms were then removed from the chambers and permitted to recover for 48 hours, at which time the number of surviving worms on each plate was determined by Automated Wormscan.

## Results

### Automated Wormscan implementation and operation

The required software for Automated WormScan can be downloaded as an easy to install complete software package available at doi, 10.5256/f1000research.10767.d152697 ^19^. Installation and running of the software are fairly intuitive, but detailed instructions can be found in the comprehensive user guide ( Supplementary File 1). The software is based on the FIJI image analysis software package, version 1.49m. If FIJI is already installed, the plugins and macros can be added to the current installation, as described in the user guide ( Supplementary File 1). A macro designed to work with Microsoft Excel is also provided to assist with data compilation and formatting. The software has been developed to run on a computer running Windows 7 or higher. The minimum system requirements are low, with the program confirmed to run on a desktop PC with an Intel Core 2 processor and 4GB ram.

Automated Wormscan source code Click here for additional data file. Copyright: © 2019 Puckering T et al. 2019 Data associated with the article are available under the terms of the Creative Commons Zero "No rights reserved" data waiver (CC0 1.0 Public domain dedication).

A set of twelve 6 cm plates are scanned as described in Methods and in the user guide ( Supplementary File 1). Computational analysis of the images begins with the flipping of the images around the vertical axis to make the plate locations correspond intuitively to their locations on the scanner. This image is then split into 12 individual images corresponding to each individual plate. The second scanned image of the same 12 plates is likewise split into 12 individual images. Each of the 12 individual images from the first scan is paired with the corresponding images from a second scan. These paired images are then precisely aligned using image stabilization (Kang Li. 2008. The image stabilizer plugin for ImageJ. Available: http://www.cs.cmu.edu/~kangli/code/Image_Stabilizer.html) so that each pixel can be compared for a difference in intensity between the two images. The outcome is a greyscale difference image, in which the whiteness intensity of each pixel corresponds to the degree to which that pixel differed in intensity between the first and second scan. This is then converted to true black-and-white with the use of an edge detection function (Boudier T, Meys J. 2015. Edge Detection. Available: http://imagejdocu.tudor.lu/doku.php?id=plugin:filter:edge_detection:start). Particle analysis of this image is then performed, which generates a count of areas on the image that differ between the two scans, as well as circumference measurements for each of these. Size parameters are pre-set to exclude identified areas that are too large or too small to be L4 nematodes (>6mm, <0.4mm in diameter), such as refractive areas of the petri dish or dust particles. It is possible to alter the pre-set parameters to identify and count worms of other developmental stages.

**Figure 1.**
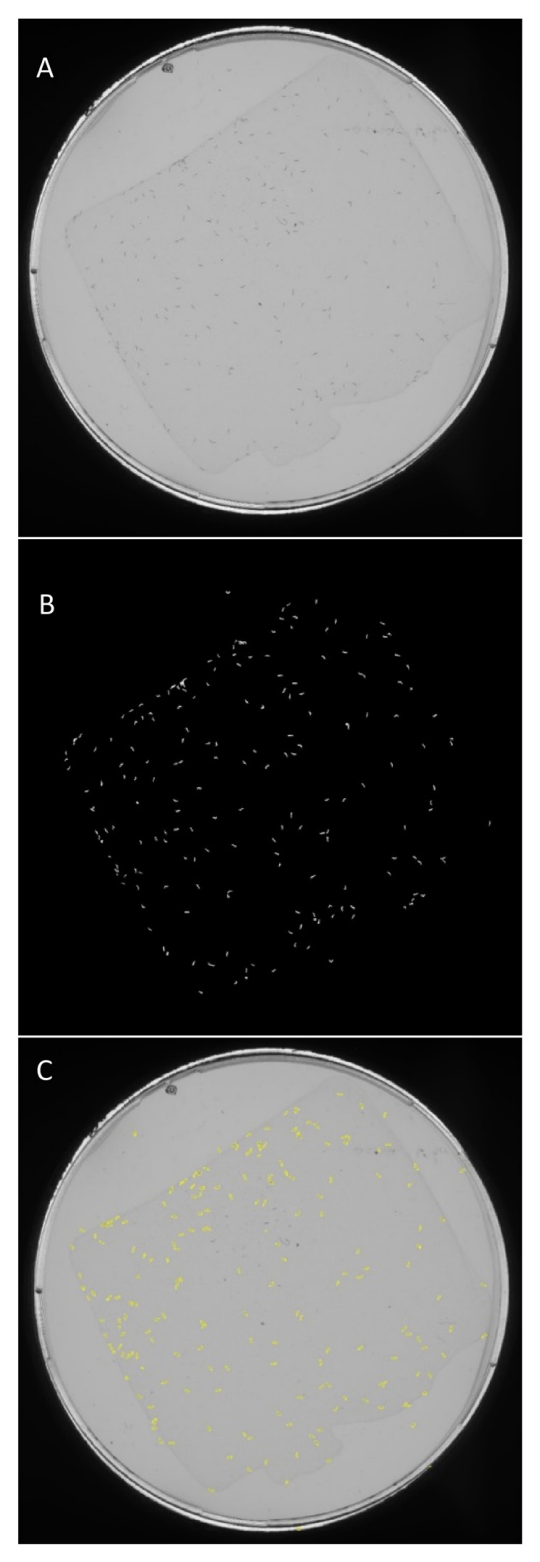
Movement based identification of *Caenorhabditis elegans* based on pixel intensity difference between two successive scans of an agar petri dish. ( **A**) A high-resolution scan of a petri dish containing a population of approximately 100 early L4 stage *C. elegans* individuals. ( **B**) A difference image produced from a pixel-by-pixel comparison with a second image taken of the same plate ten minutes later. White regions correspond to a worm that moved from its original location during this time. ( **C**) Regions of interest from the difference image that have worm-like size and shape attributes are identified. Archival images are created with an outline of identified worm-like objects overlaid on the original images.

The worm identification, measurement and counting functions are fully automated, so will proceed until an entire folder of scanned images has been processed. A difference image with worm-like features highlighted is created for each plate and is also archived to allow visual confirmation of results and as a permanent record of the experiment ( Figure 1). The processing of a large number of scans (>30) requires several hours. However, a human operator is not required during this entire period, so it is convenient to run the process overnight.

**Figure 2.**
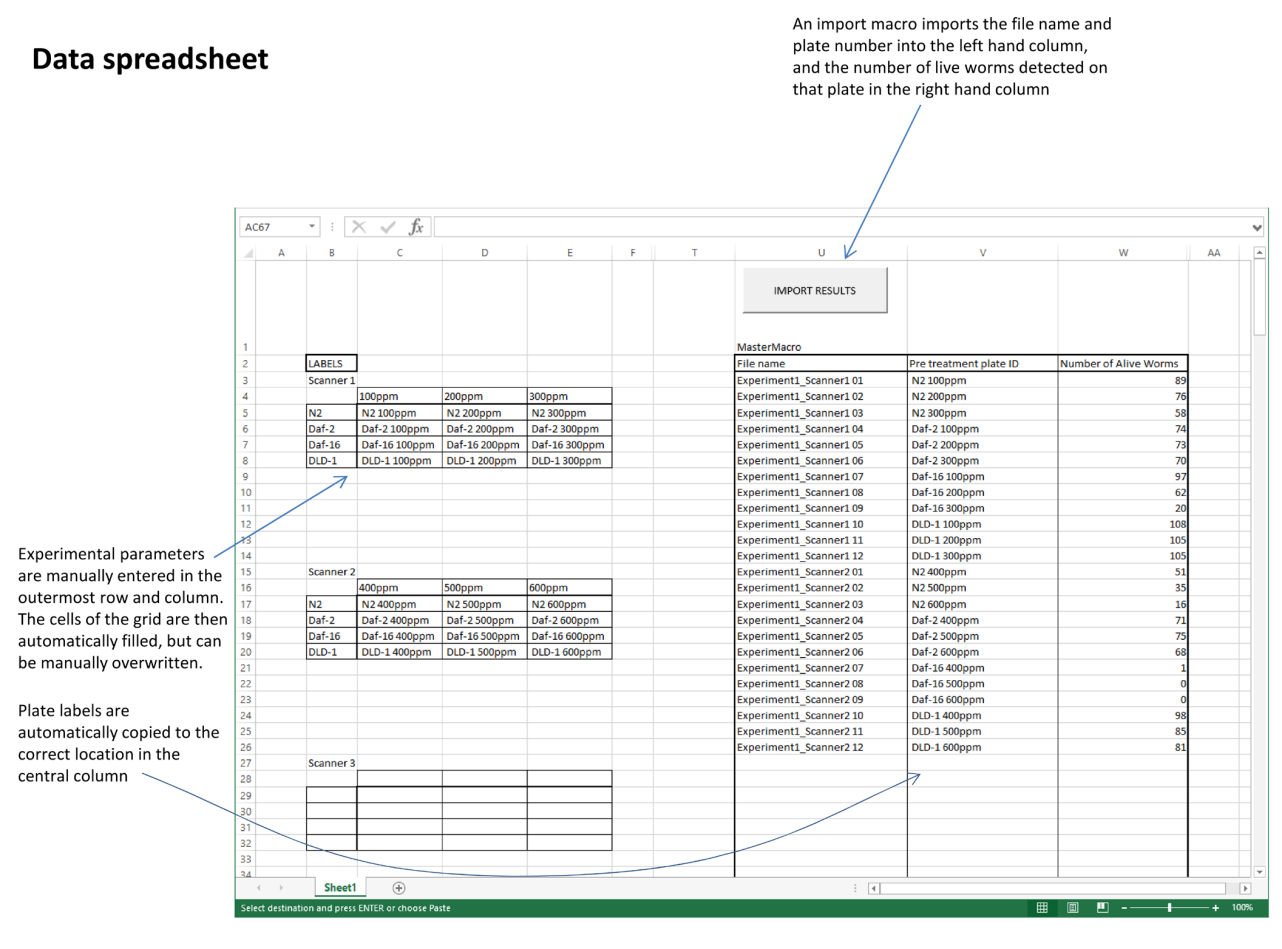
Example data-processing spreadsheet. A count of live worms from every scanned petri plate is extracted from the output of the FIJI plugin and is associated with its scan number and plate position. Researchers then manually enter experimental parameters to automatically fill the 3x4 grids with presumed plate labels by an auto-completion function. If the design of an experiment does not fit this default model, the labels can be inserted manually. The end result is a single table of raw data from the entire collection of images that can easily be tracked back to the archived images.

The end product of the computational analysis is a spreadsheet for each individual plate, containing a series of measures of each area of difference (and hence, of each worm). These measurements include circumference, area and circularity, which are potentially useful for comparing worm size or developmental stage. An Excel macro has also been developed, which imports the data from the FUJI plugin into an Excel spreadsheet template ( Supplementary File 2). This results in a table of raw data suitable for statistical analysis and graphing ( Figure 2).

### Assays demonstrating the utility of the system

***Accuracy and consistency of Automated WormScan relative to counting by humans.*** The reliance of Automated WormScan on comparing the worm count prior to an experiment to the worm count after an experiment, simplifies the computational burden relative to employing machine learning to identify and assess mortality of worms from a single pair of post-experiment scans. While our procedure makes worm identification extremely robust, errors associated with both the pre- and post-experiment counts contribute to the final error of the analysis. This differs from human counting, as well as from the machine learning procedure originally described in WormScan and utilised by the Lifespan Machine, both of which count live and dead worms from a single set of scans.

**Figure 3.**
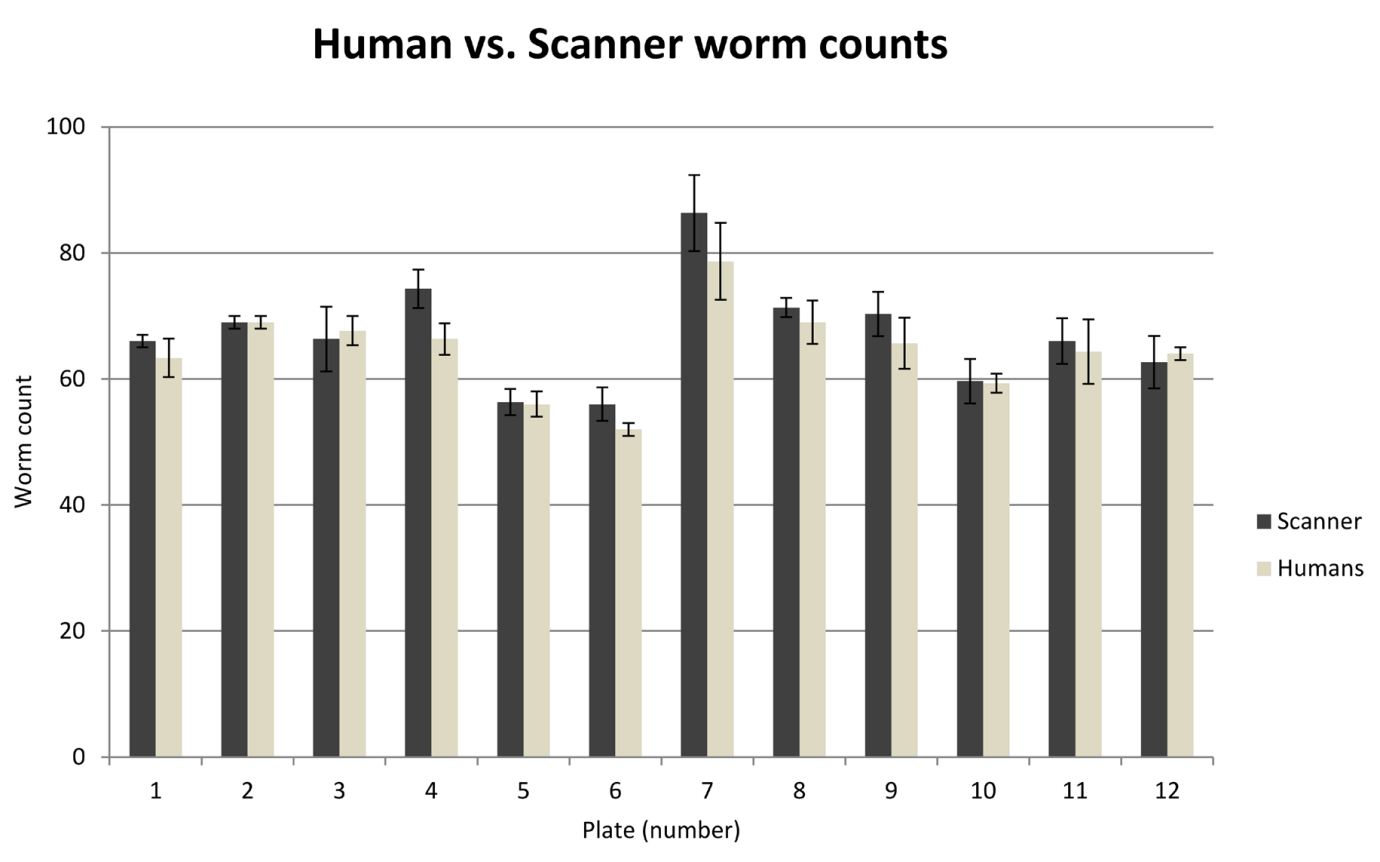
Comparison of worm counts produced by three different human observers *vs* three independent scans. Variability in numbers of individual worms counted by Automated Wormscan on the same plate is not significantly different from the variation in counts between three independent human observers using traditional methods. Error bars indicate standard deviation over three independent counts.

To establish the error rate of the automated system, an assay was performed to count the number of live worms on agar plates using both traditional human counting and Automated Wormscan. In total, 12 plates of worms were counted in triplicate using a microscope, each time by a different trained human. The same plates were then scanned sequentially three separate times, each time in a different scanner, with counts performed using Automated WormScan ( Figure 3). We performed a Generalised Linear Mixed-Effects Model ^20^ in R (version 3.3.2) ^21^. Overall, we found that counts produced by human observers *vs* counts produced by Automated Wormscan did not differ significantly (P>0.2), indicating that Automated Wormscan closely replicates human counting.

**Figure 4.**
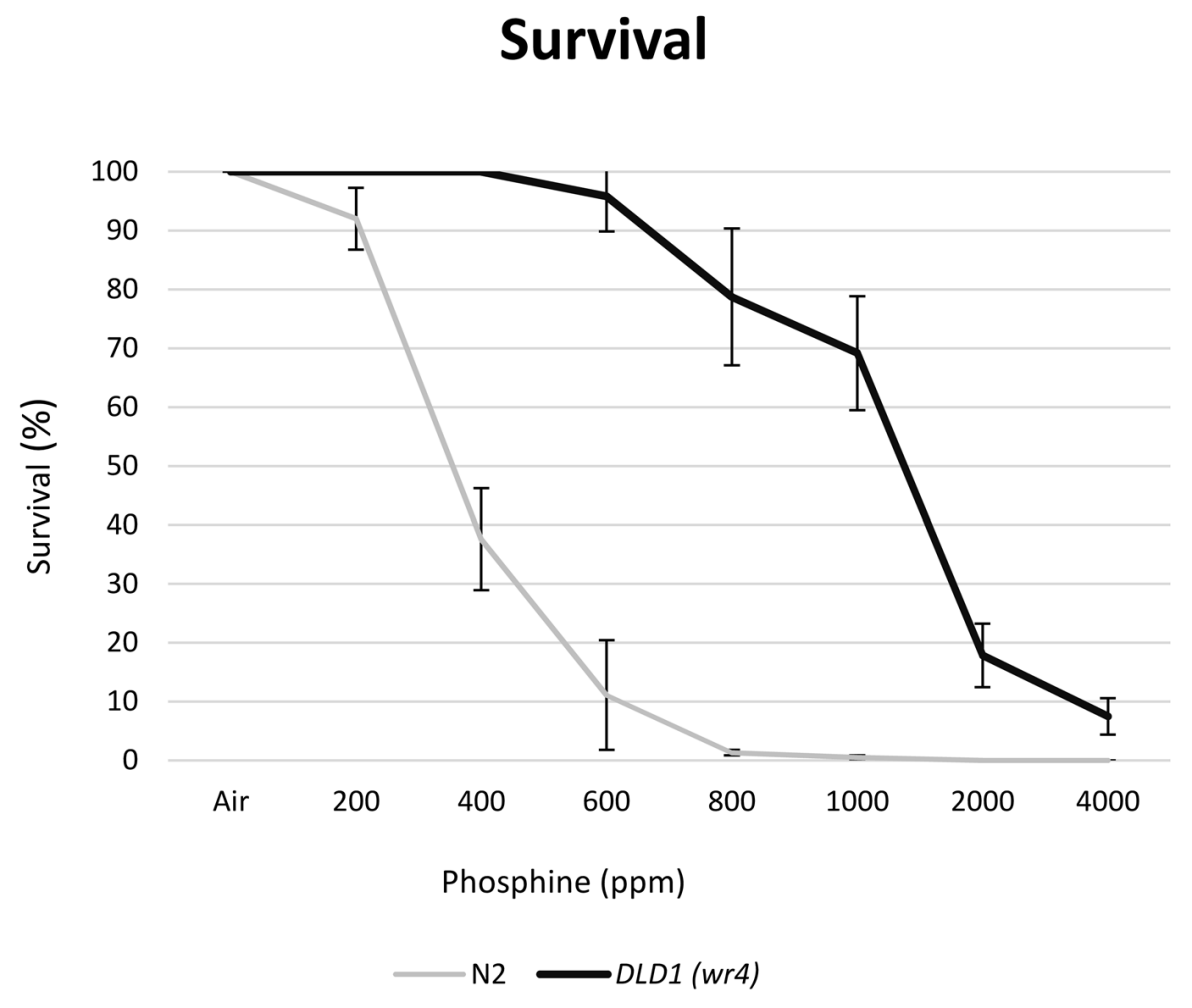
Phosphine mortality curves of wild type and resistant strains of *Caenorhabditis elegans*. Automated Wormscan accurately duplicates previously published phosphine toxicology results. Wild type worms (N2) display an LC _50_ of ~400ppm, while the phosphine resistant mutant *dld-1(wr4*) display an LC _50_ of ~1800ppm, congruous with recently published results ^6^. Error bars indicate standard deviation over three experimental replicates.

***Wormscan is able to replicate previously published toxicological data.*** We replicated phosphine exposure protocols used by our laboratory to demonstrate that Automated Wormscan generates results consistent with published results derived from manual counting. Depending on the dose of exposure, toxins can impair mobility or alter the shape of the worms, but this does not materially affect the outcome of the analysis. We exposed wild type N2, and a phosphine resistant strain, *dld-1*( *wr4*), to a range of phosphine concentrations and obtained survival curves indicating LC _50_ values of 400ppm for N2 and 1800ppm for *dld-1.* These results, demonstrating a phosphine survival rate for *dld-1*( *wr4*) 4.5 times greater than wild type, are consistent with a previously published study that used manual phenotype scoring, which found the survival rate of *dld-1*( *wr4*) to be 4.4 times greater than wild type ( Figure 4) ^16^.

**Figure 5.**
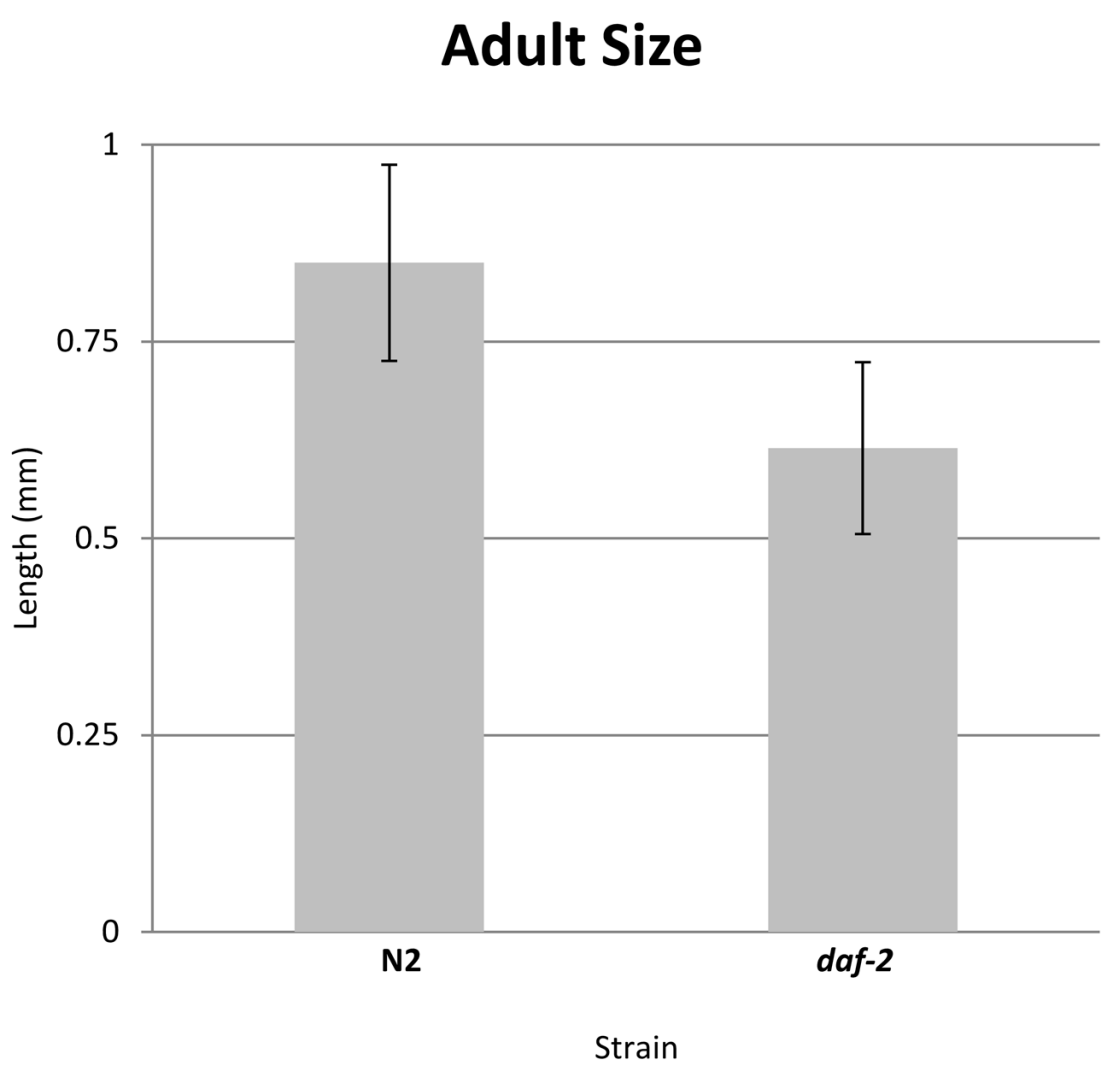
Growth rate of *daf-2* worms compared with wild type. Length was calculated as ½ of the perimeter measurement determined by Automated WormScan. Error bars represent standard deviation over three experimental replicates.

***Automated wormscan can be used to measure growth rate and fecundity.*** While counting living, mobile and stimulus responsive worms is the primary objective of the system, the perimeter of each identified worm-like object is also determined during analysis. We therefore performed an assay to determine how well this data could reproduce published size difference of two different worm strains, N2 and *daf-2*, as *daf-*is well known to have a slow growth rate and delayed maturation relative to the wild type N2 strain ^22, 23^. Plates were scanned 48 hours and 90 hours after seeding with synchronised L1 worms. After 48 hours, no progeny were present on any of the plates, and the average perimeter data was recorded for each worm and length was calculated as 1/2 × perimeter. Using Automated WormScan, *daf-2* worms were determined to be significantly shorter than wild type worms after 48 hours growth at 20°C, by an average of ~0.25mm ( Figure 5). This reflects the well-established size difference of the *daf-2* strain.

**Figure 6.**
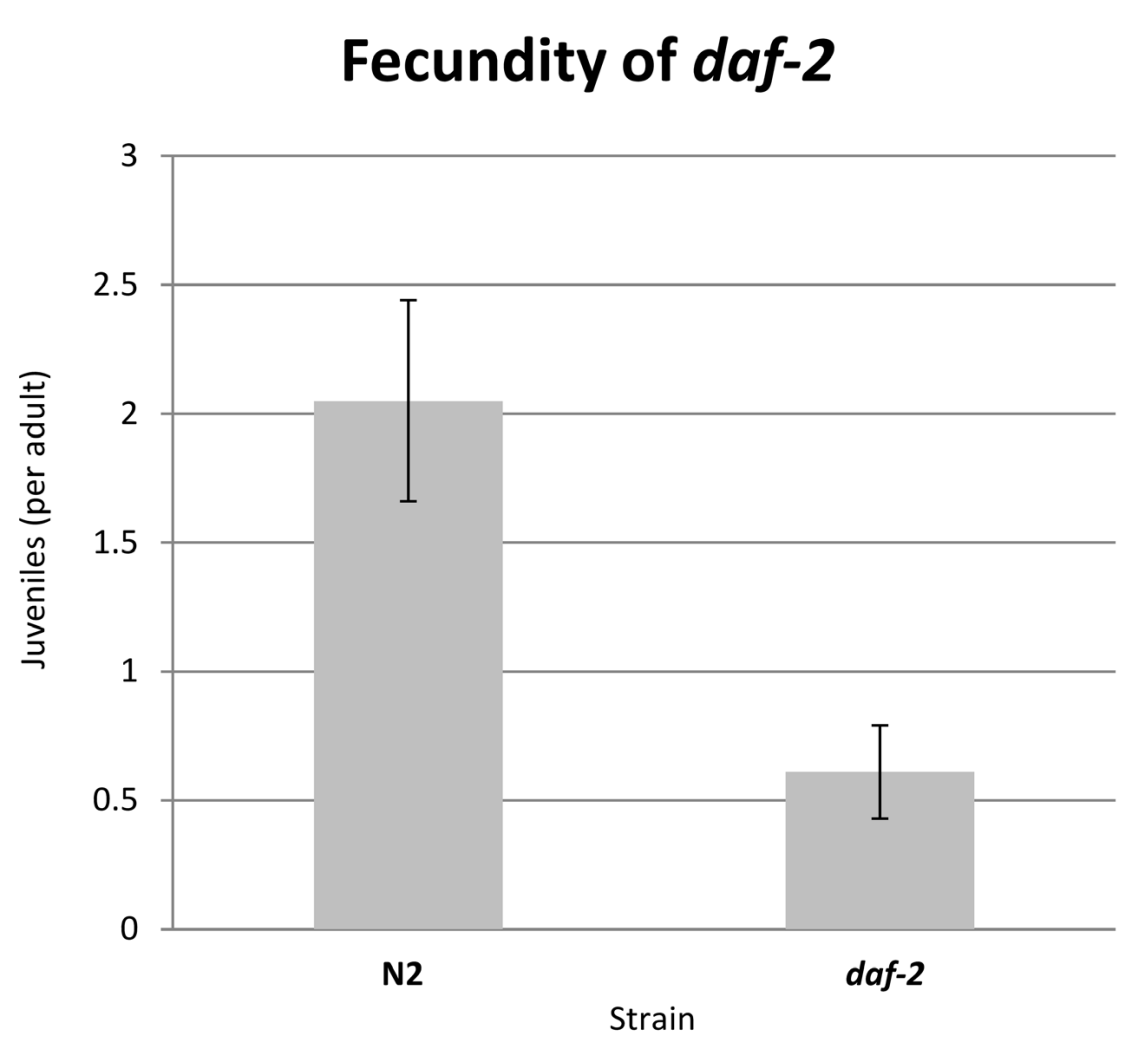
Reduced fecundity at 90 hours of *daf-2* worms compared to wild type. Fecundity is expressed as juvenile-sized objects (<0.5mm perimeter) per adult-sized object (>1.5mm perimeter) as counted by WormScan. Error bars represent standard deviation over three experimental replicates (>600 juvenile individuals scored).

After 90 hours at 20°C, both strains had produced progeny that were easily recognised by their size, with adults being well over 1.5mm in perimeter and juveniles being under 0.5mm. The rate of reproduction of each strain is presented as juveniles present per adult worm ( Figure 6). The developmental stage of each individual was confirmed visually on the source image. After 90 hours, wild type worms produce approximately 4 times as many progeny as *daf-2* worms, which is consistent with the well-established decrease in reproduction of the *daf-2* strain.

***Accuracy of automated wormscan using compressed image formats.*** We find that it is essential to scan at high resolution to obtain accurately identified worms, but this produces TIFF format image files of individual petri plates of ~60 MB. For the small experiment shown in Figure 4, the 14 data points were generated from 3 experimental replicates, each of which contained 2 technical replicates. As each plate is scanned a total of 4 times, a total of 336 images were generated. Given a TIFF image size of 60 MB, the total storage requirements if using high resolution images would be 20 GB. High resolution images also increase the computational processing demands.

Therefore, we carried out an experiment to see if the performance of our system was compromised by use of compressed image formats. A series of plates of N2 worms in the early L4 stage of development were analysed by Automated WormScan in two different ways. In both cases, the worms were initially scanned as 16 bit greyscale images. In the first case, the images were saved in the lossless TIFF format (~60 MB) by the scanning software. In the second case, the images were saved as compressed high quality jpg images (~1.5 MB) by the scanning software. These images were then processed by Automated WormScan to compare the number of worms counted per plate in each image format. We found that analysing compressed jpg images had no effect on the number of worms that could be identified by the program ( Supplementary File 3).

Raw worm counts for Figure 3 - comparison of worm counts produced by three different human observers vs three independent scans Click here for additional data file. Copyright: © 2019 Puckering T et al. 2019 Data associated with the article are available under the terms of the Creative Commons Zero "No rights reserved" data waiver (CC0 1.0 Public domain dedication).

Raw worm counts for Figure 4 - phosphine mortality curves of wild type and resistant strains of Caenorhabditis elegans Click here for additional data file. Copyright: © 2019 Puckering T et al. 2019 Data associated with the article are available under the terms of the Creative Commons Zero "No rights reserved" data waiver (CC0 1.0 Public domain dedication).

Raw worm counts for Figure 5 - growth rate of daf-2 worms compared with wild type Click here for additional data file. Copyright: © 2019 Puckering T et al. 2019 Data associated with the article are available under the terms of the Creative Commons Zero "No rights reserved" data waiver (CC0 1.0 Public domain dedication).

Raw worm counts for Figure 6 - reduced fecundity at 90 hours of daf-2 worms compared to wild type Click here for additional data file. Copyright: © 2019 Puckering T et al. 2019 Data associated with the article are available under the terms of the Creative Commons Zero "No rights reserved" data waiver (CC0 1.0 Public domain dedication).

## Discussion

Automated Wormscan is an extremely low cost system (scanner USD$500, 3TB internal hard drive $125, hard drive dock $25, scanner plate holder $50, standard PC with Windows operating system) capable of replacing manual counting of worm populations and scoring of survival. We have demonstrated that the system is capable of replicating previously published toxicological results, while also yielding greatly improved speed and throughput. The system is remarkably easy to set up, requiring less than 60 minutes. Once set up, very little operator time is required to carry out the image acquisition and analysis. An archival record is automatically stored as a compressed image file on which identified worms are highlighted. Accuracy of record keeping is enhanced by the ability to collect all image files for an experiment in a single folder and process them all at once, with the output being a single Excel spreadsheet of data with filenames and plate numbers transferred directly from the image files.

*C. elegans* toxicological assays are often limited to only a handful of strains, treatments, time points and/or chemical concentrations, due to the inability of researchers to quickly score very large numbers of individual worms. The use of an automated mortality scoring system removes this significant bottleneck in the throughput of most toxicological assay designs. This greatly improves the speed at which such experiments can be performed and enables complicated experiments to be contemplated, such as the simultaneous determination of the combinatorial effects of many substances at a variety of concentrations. Furthermore, the increased throughput enables the use of larger numbers of technical replicates and extra experimental replicates, which provides greatly improved statistical power that previously would have been impractical due to the labour required.

Automated WormScan is one of several systems recently developed that utilise image capture and computer-based phenotype assessment to improve the throughput or accuracy of such *C. elegans* assays ^1, 2^. Automated WormScan uses very inexpensive and simple hardware, requiring only a basic desktop PC and at least one Epson v700 (or v800) scanner and a plate holder to align petri plates on the scanner (LabPro Scientific; part LPST12-1). Automated WormScan requires no modifications or alterations to the scanners and the software is also especially easy to install and simple to use. Setup can be accomplished in under an hour by anyone with basic computer skills. In addition, scans are taken while the worms are on an NGM agar plate, which is the typical format for most *C. elegans* assays. This means the scanning can be incorporated into existing experimental methods without requiring alterations to the experimental procedure.

Since the system focuses on the ability of the animals to move under stimulus, the assay is very robust, with a demonstrated ability to consistently count live worms with the same accuracy as a human. The robustness of the system is achieved largely by avoiding the machine learning steps integrated into the primary component of WormScan, in favour of the simpler difference image analysis method that was originally developed for monitoring survival in lifespan experiments ^1^. This design decision increases the amount of handling required compared to the machine learning strategies of the original WormScan and Lifespan Machine systems ^1, 2^ (i.e. two pairs of scans must be taken instead of one). In practice, this requirement is not onerous. Automated Wormscan is not capable of resolving multiple worms in close contact. In practice, this is not usually a problem, as clearly demonstrated in Figure 1. The solution is simply to use ≤100 worms per plate, in which case the simplicity of the Automated WormScan software does not significantly impinge on the ability of the system to replicate human counting. Automated WormScan will not work with mutants or experimental conditions that induce aggregation of worms, but the same is true of the other counting systems and probably human counting as well.

Automated WormScan is time and labour efficient, requiring ~10 minutes of operator time per 60 plates (5 scanners) and 20 minutes of scanning time (during which an operator need not be present). Once all scans have been completed and saved to a single folder, another 5 minutes of operator time is required to start the analysis software. The software requires about 30 seconds to analyse each pair of images (but this requires no operator involvement). Thus 6,000 individual *C. elegans* (at a density of 100 per plate) can be analysed with 15 minutes of operator time and about 60 minutes of elapsed time *.* The total capacity of the system is limited only by the number of scanners that are used in parallel to generate the source images, so the system can be scaled to suit the requirements of the user.

User bias in methods that involve manual counting is a known source of data variation ^24^, especially between institutions ^25^. The use of this automated method eliminates this possibility while simultaneously improving consistency. Furthermore, the use of the light stimulus obviates the need to open the plates and physically touch the animals, which minimises the potential for contamination and removes the possibility of physically damaging the animals.

While the lifespan machine utilises modified scanners to reduce temperature fluctuations, the focus of Automated WormScan for mortality based toxicology assays means that worms do not spend much time in the environment of the scanner and are able to be housed in temperature controlled conditions for the great majority of time, obviating the need to modify the scanners to compensate for temperature. Also, while Lifespan Machine requires modifications to the scanner to adjust the instruments focal plane, the robustness of difference imaging produces accurate counts of worm populations using entirely unmodified scanners.

Since compressed image formats do not hamper the ability of Automated WormScan to accurately quantify *C. elegans* populations, we utilise compressed .jpg files, rather than the much larger TIFF images utilised by other systems ^1, 2^. This obviates the need for greater than terabyte data storage for most applications, and brings the system specifications required to run previously computer resource intensive image processing phases into the range of a standard modern desktop PC.

## Software and data availability

Dataset 1. Automated Wormscan source code: doi, 10.5256/f1000research.10767.d152697 ^19^

License: GNU General Public License

Dataset 2: Raw worm counts for Figure 3 - comparison of worm counts produced by three different human observers *vs* three independent scans. doi, 10.5256/f1000research.10767.d152698 ^26^

Dataset 3: Raw worm counts for Figure 4 - phosphine mortality curves of wild type and resistant strains of *Caenorhabditis elegans.* doi, 10.5256/f1000research.10767.d152699 ^27^

Dataset 4: Raw worm counts for Figure 5 - growth rate of *daf-2* worms compared with wild type. doi, 10.5256/f1000research.10767.d152700 ^28^

Dataset 5: Raw worm counts for Figure 6 - reduced fecundity at 90 hours of *daf-2* worms compared to wild type. doi, 10.5256/f1000research.10767.d152701 ^29^

## Supplementary material

**Supplementary File 1: Automated Wormscan hardware and software installation and setup guide.** A step-by-step guide to installing and setting up all hardware and software necessary to run Automated Wormscan. Includes troubleshooting information.

**Supplementary File 2: Automated Wormscan Excel Mastersheet.**

**Supplementary File 3: TIF scans *vs* JPG scans.** Data is live worm counts produced by Automated Wormscan from 12 plates scanned first in JPG and then in TIF file format.

**Supplementary File 4: Template for making a scanner guide.**
