# Supplementary material for: Automated Wormscan
Source: F1000Res. 2019 Jan 4;6:192. Originally published 2017 Feb 27. [Version 3] doi: 10.12688/f1000research.10767.3 (PMC5365223; doi:10.12688/f1000research.10767.3)
Supplement: Supplementary file 6 [file f1000research-6-19096-s0005.tgz › 4aa2a498-b5db-46cb-9beb-c889740c5fdf.docx]

**Setup guide**

1: Download the Automated Wormscan source code at doi, 10.5256/f1000research.10767.d152697 and unzip the compressed file. This contains two further compressed files, one (Full Fiji Install.rar) contains a complete installation of the FIJI and Automated Wormscan softwares. The second (plugins and Macros (for existing FIJI installs).rar) contains the Automated Wormscan files and the files for two third party FIJI plugins on which Automated Wormscan relies.

2: Unzip the file ‘Full Fiji Install.rar’

3: With File Explorer open, click and drag the ‘Fiji’ folder directly on the C: drive (i.e. not in any subfolder on the C: drive). See below if you cannot or do not wish to place the Fiji folder directly on the C: drive, or if you already have an installation of Fiji.

4: Open the file ‘ImageJ-Win64’ (This will open ImageJ – for future convenience make a shortcut to this file at a location of your choosing).

5: Click ‘Analyze > Set Measurements’ and ensure the following options are checked:

Area Centroid Perimeter

Worm Counter is now installed.

6: Download and install the “Epson scan” software available at <http://tech.epson.com.au/downloads/category.asp?sCategory=Scanner&id=perfectionv700photo&SelOS=Y>

**If you already have FIJI installed:**

The plugins and macros that must be added to Fiji are in the folder ‘plugins and macros’

Place the following files in your Fiji/plugins directory:

Image_Stabilizer.class

Image_Stabilizer_Log_Applier.class

Hysteresis_.class

WormAnalysisProgram_.java

SingleWormPlateAnalysis_.java

Place the following file in your Fiji/macros directory:

wormscanmastermacro.ijm

Open Fiji and drag WormAnalysisProgram_.java onto the Fiji window to open it. At the very bottom of the plugin is a line that directs the program to the location of the counting macro. By default it is:

C:\\Fiji\\macros\\wormscanmastermacro.ijm

Note that double backslashes replace single forward slashes in FIJI This will need to be changed to the location of your Fiji install and saved. Repeat this process with SingleWormPlateAnalysis_.java

**You must install Fiji at a location other than the C: drive.**

All plugins and macros are already present in the copy of Fiji we distribute. However, you will need to direct the two Automated Wormscan plugins to your chosen installation location as directed above

Make a **black** plateholder to the dimensions provided (File: Scanner Plate Holder.pdf). Do not omit the cutout (empty space) at the start of the plateholder (bottom on the schematic) as this is needed for the scanner to calibrate. Also, the crowding of the plates toward the centre of the plateholder is intentional, so adhere to the dimensions in (File: Scanner Plate Holder.pdf). For simplicity, plateholders can be obtained from Labpro Scientific, Australia (part number LPST12-1) [sales@labproscientific.com](mailto:sales@labproscientific.com).

Place plates in the appropriate spaces. It is not necessary to fill every space.


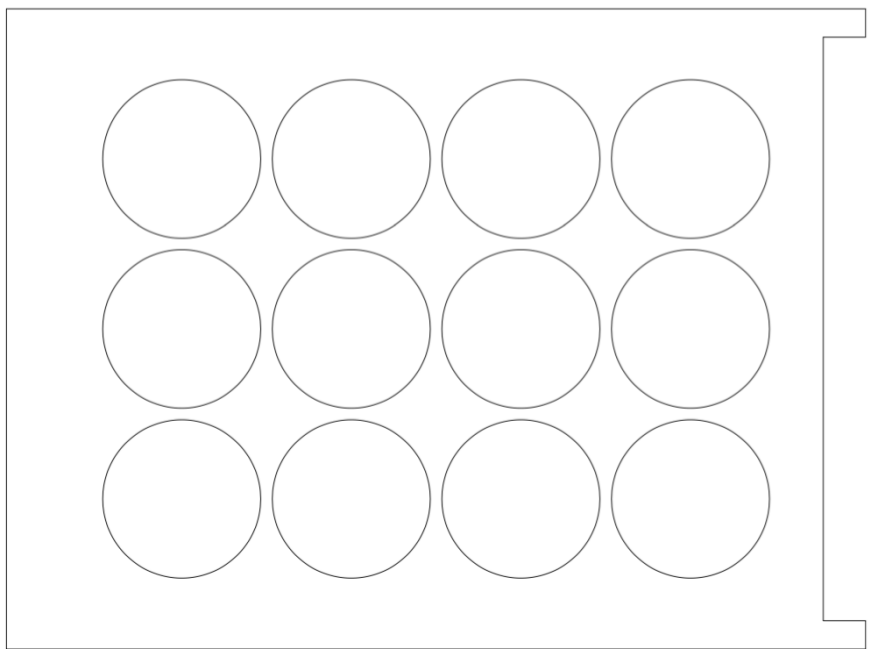


**🡪 Direction of scan 🡪**

This cutout is essential for scanner calibration

Record the plate labels of each plate, and the location each was placed. A simple table like the following is helpful:

Experiment:

Scan number:

Filename:

|  |  |  |
| --- | --- | --- |
|  |  |  |
|  |  |  |
|  |  |  |

**Scanning guide**

Ensure the scanner is plugged in and switched on before opening the Epson scan software.

Ensure the Epson scan software is set up exactly according to the following screen capture:


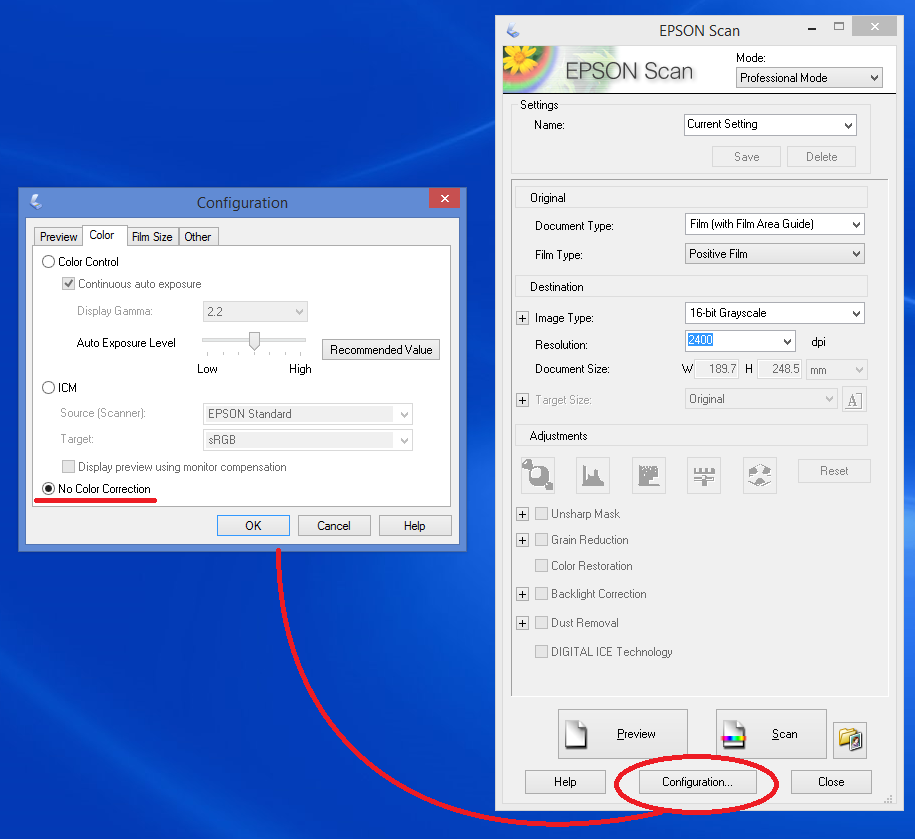


Click ‘preview’ and check that the image is satisfactory.

To set the scanner to take 2 consecutive scans:

1: Draw a selection box on the preview scan encompassing all 12 plate locations, regardless of whether they are filled.

2: Click the ‘duplicate’ button

3: Drag the resulting second box so that it **precisely** overlaps the first

4: Click ‘All’ to select both marquees at once.


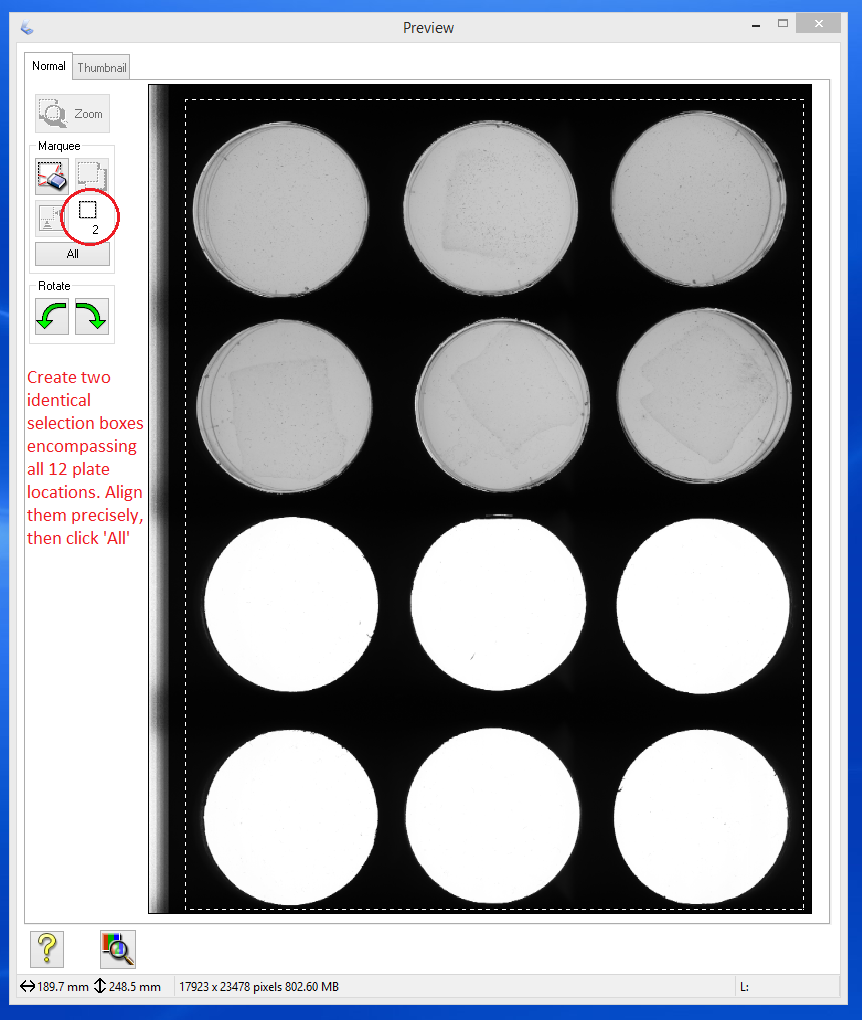


This will prime the scanner to take 2 consecutive scans.

Click ‘scan’.


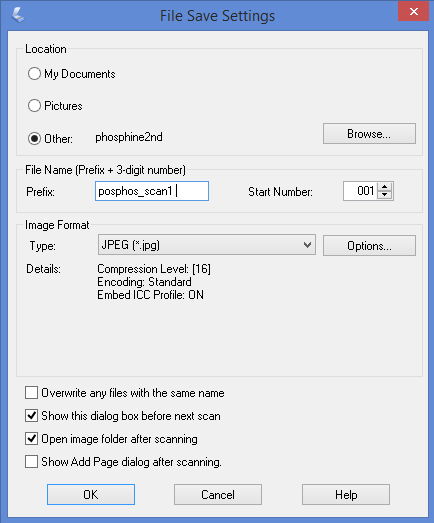


Ensure the file type is set to JPEG.

**Your file name must be a single string (no spaces) of alphanumeric characters followed by a single space.**

Set the scan number to 000

This will ensure that the image file names are in the format of a single string of characters followed by a space, followed by the numbers 000 and 001 respectively for the two sequential scans. This is necessary for the program to work.

Note: If the filenames contain the letters ‘pre’ or ‘post’, then the excel spreadsheet will automatically place data from images labelled ‘pre’ next to images labelled ‘post’. For example, if a pre-treatment scan is labelled ‘James_Experiment1_pre ’ and its corresponding post-treatment scan is labelled ‘James_Experiment1_post ‘, the results from these scans will be placed side by side in the final spreadsheet. If you do not wish to use this feature, avoid the use of ‘pre’ and ‘post’ in your file names.

If using multiple scanners:

We have found that the best way to run multiple scanners is to set up a windows user profile for each scanner. Once a scan is started for the first scanner using the first profile, switch to the next profile and initiate scanning with the second scanner using the same procedure that you used for the first scanner. Scanners associate themselves with the profile that is active when the scanner software is first run. This association can be reset by unplugging the scanners from the PC and restarting windows. High resolution scans of twelve 6cm plates takes about 10 minutes (20 minutes for 2 sequential scans). One person can easily operate more than 5 scanners at a time.

**Automated Wormscan running guide**

Click ‘Plugins > WormAnalysisProgram’

Set your hysteresis and size exclusion settings. The program defaults should be sufficient for L3, L4 and adult *C. elegans*.

If your analysis requires non-standard settings . . .

The level at which hysteresis is set determines the sensitivity of detection, while the difference between the high and low setting influences the threshold at which two regions of interest are considered separate. Lowering the hysteresis settings will result in the program being more sensitive to movement, but also increases the likelihood that it may pick up mobile dust, fibre, condensation, or worm trails. Conversely, raising them may help the program ignore such objects while increasing the possibility that smaller or less active worms may be overlooked. **Note: condensation on the petri dishes, overly thick media, or overly thick bacterial lawns can produce blurry scans. Lowering the hysteresis settings to 15 high / 10 low, or even as low as 8 high / 5 low can help the program produce an accurate count of such a plate.**

The size exclusion settings determine the minimum and maximum size of mobile objects that are to be considered as worms. The default settings exclude only objects a few pixels in size. An example of when it may be useful to change this setting is if there are unwanted juveniles on the plates. Raising the minimum allowable size would help exclude these from consideration.

The precise range at which hysteresis and size exclusion operate will differ depending on experimental conditions. Some trial and error may be needed for plates that diverge from standard conditions. The individual plate counting plugin is useful for this purpose (see below).

Click OK, and you will then be asked to select a folder in which the scanned images are held. This folder should be located on the main drive of the computer running Automated WormScan (i.e. not on thumb drives or external drives). **The folder should contain scans only.**

The program will now run. It is recommended that the computer not be used for any other purpose during this time.

On completion, the folder that held the original scans will now contain 3 sub-folders. ‘Results’ will contain an excel spreadsheet for each plate with the worm count for that plate and the perimeter data for each worm. ‘Single Images’ contains the images of each plate cut out from the original scan. These can be used if desired to re-count using different settings without having to run the entire macro again (see below for instructions). ‘Movement Comparison’ contains TIFF stacks with a region of interest overlay. These are useful to review the program’s counting decisions by eye.

Note: if a user wishes to run the entire count again on the same images, these sub-folders must be deleted, or the original scans must be copied to a new folder. The program can only be run on a folder that contains only scans.

**To re-count one or more individual plates after the first run of the program:**

Users may wish to re-count one or more individual plates using different settings. Rather than re-running the entire Worm Counter plugin, the ‘SingleWormPlateAnalysis’ plugin can be used. Place the image pairs that are to be recounted in their own folder. It is fine to include multiple image pairs, or pairs from different scans, provided that the two images for each plate are both present. Run the ‘SingleWormPlateAnalysis’ plugin. Results will be generated in the same fashion as the main plugin.

**Excel Mastersheet guide**

Open ‘Wormscan Mastersheet.xls’. (Supplementary File 2) This is write-protected, so immediately save a copy under a new filename associated with the experiment. Once it is saved, you will be able to manipulate the new copy of the Mastersheet.

Ensure macros are enabled (excel will give a warning if this is not the case).

Click the ‘import results’ button.

Direct the program to the ‘results’ folder of a completed Automated Wormscan process.

The filenames and mobile worm counts of all the plates from that folder will be imported in alphanumeric order of the filename of each scan, and within each scan from top left plate to bottom right plate. Microsoft Excel treats a number within a name as text. As a result, ordering of names may be counterintuitive. For example, results from images from a scan labelled ‘experiment1scan10’ will be listed before results from a scan labelled ‘experiment1scan1’. It may be necessary to account for this when entering plate labels (e.g. use “experiment1scan01” instead of “experiment1scan1”.)

If the string ‘pre’ or ‘post’ was included anywhere in the filename, scans will be sorted appropriately to the pre-treatment and post-treatment columns of the Wormscan Mastersheet. The plate labels are then manually entered into the tables provided on the left of the spreadsheet. If the plates were organised systematically, you can use the outermost boxes to autocomplete the cells of the table. In the example, the strain names were entered in the left hand column and treatments were entered in the topmost row. The individual cells of the chart were then automatically filled. The labels from the table are automatically copied to the data spreadsheet. With sensible organisation of plates in the scanner, the autofill functions of the Excel Mastersheet can dramatically simplify labelling and decrease the possibility of inadvertent errors.
