# Supplementary figures and images for: Automated Wormscan
Source: F1000Res. 2019 Jan 4;6:192. Originally published 2017 Feb 27. [Version 3] doi: 10.12688/f1000research.10767.3 (PMC5365223; doi:10.12688/f1000research.10767.3)

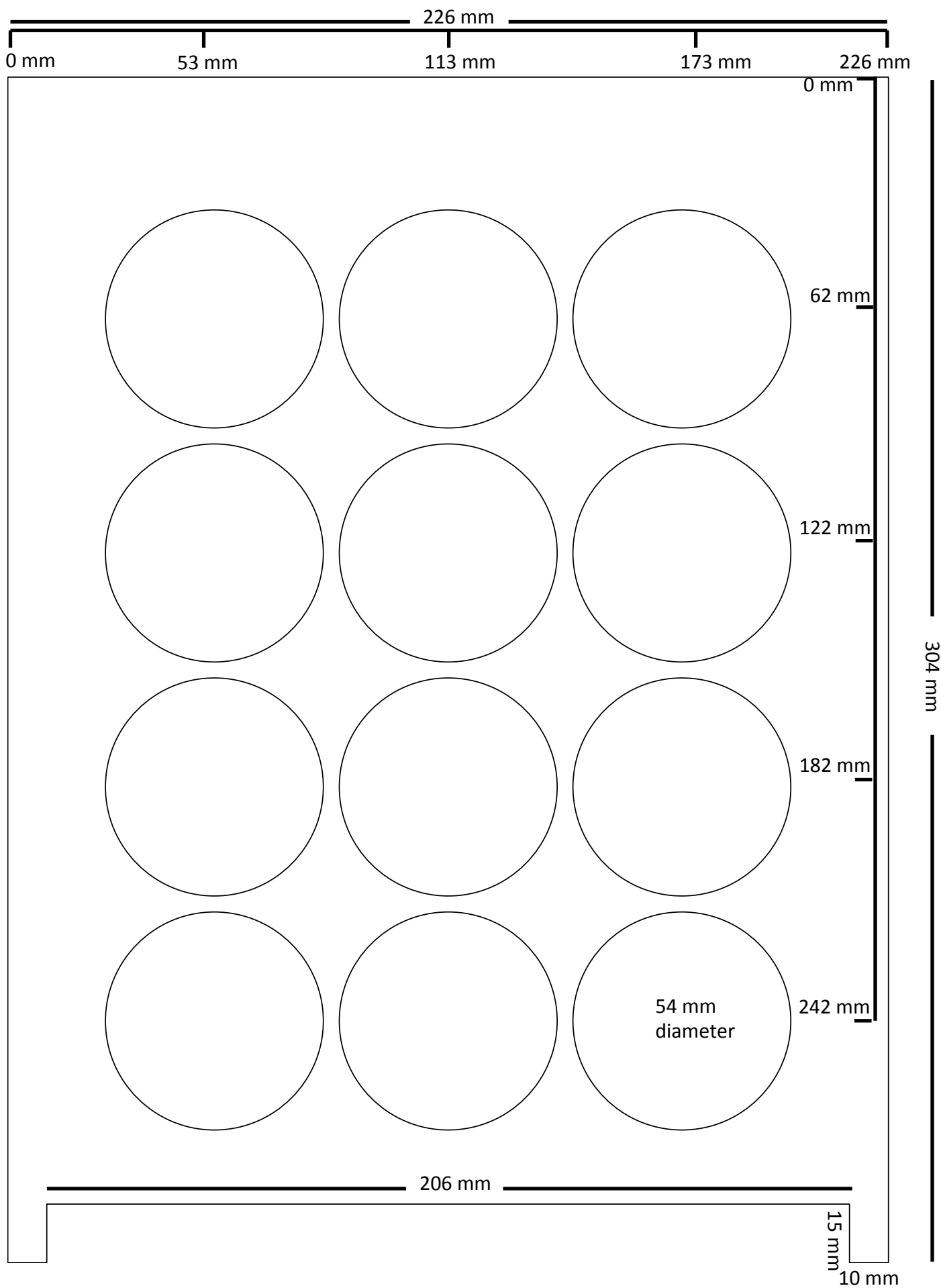

Supplement: Supplementary file 9 [file f1000research-6-19096-s0008.tgz › a93fbe43-69d0-406b-a802-d80ee0e78b38.pdf]
