# Supplementary material for: Automated Wormscan
Source: F1000Res. 2019 Jan 4;6:192. Originally published 2017 Feb 27. [Version 3] doi: 10.12688/f1000research.10767.3 (PMC5365223; doi:10.12688/f1000research.10767.3)
Supplement: Peer review history

# Peer review: Referee response for version 3

**DOI:** 10.5256/f1000research.19096.r42550

**Published:** 2019-01-18

**Authors:** Nathaniel Szewczyk

**Affiliations:** 1 MRC‐ARUK Centre of Excellence for Musculoskeletal Ageing Research, University of Nottingham, Nottingham, UK

According to the summary of revisions only a correction to the funding acknowledgement was added, therefore I do not feel any further review is required as the revisions don’t address my previous review.

I have read this submission. I believe that I have an appropriate level of expertise to confirm that it is of an acceptable scientific standard, however I have significant reservations, as outlined above.

# Peer review: Referee response for version 2

**DOI:** 10.5256/f1000research.13593.r25727

**Published:** 2017-09-15

**Authors:** Nathaniel Szewczyk

**Affiliations:** 1 MRC‐ARUK Centre of Excellence for Musculoskeletal Ageing Research, University of Nottingham, Nottingham, UK

According to the summary of revisions only a supplemental material was added, therefore I do not feel any further review is required as the revisions don’t address my previous review.

I have read this submission. I believe that I have an appropriate level of expertise to confirm that it is of an acceptable scientific standard, however I have significant reservations, as outlined above.

# Peer review: Referee response for version 1

**DOI:** 10.5256/f1000research.11609.r20572

**Published:** 2017-03-21

**Authors:** Courtney Scerbak

**Affiliations:** 1 Departments of Biology and Chemistry, Earlham College, Richmond, IN, USA

The ability to automatically count live *C. elegans *on agar plates accurately, in a time-efficient manner, and with relatively low cost is of great utility to many *C. elegans *researchers. This manuscript and its supplementary materials provide an open access tool to allow researchers to do just that. I especially appreciate the discussion of the benefits of keeping an archival record (of images and data) in the reproducibility of scientific research. Overall, the authors describe the use and demonstrate the accuracy of this tool well enough to allow other groups to troubleshoot its application to their particular needs.

 With some more clarification in the manuscript, I believe this manuscript will have an even greater practical impact on many *C. elegans* research groups. Below are a list of suggestions and questions:

  

1. In the Introduction, you should mention or at least refer to more techniques that are currently available for automated *C. elegans *handling and data collection. For example, see the WormBook chapter on Microfluidics: http://www.wormbook.org/chapters/www_microfluidics/microfluidics.html
2. When referring to Matthews *et al.* (2012) in the Introduction, it would be worthwhile to describe in more detail their findings that the light of the scanner (a) works to detect mortality and (b) habituates the worms to the same degree as using a platinum pick. This would address any concerns about potential detrimental health effects of the scanner light right in the introduction.
3. Is there a reason you use this particular scanner (Epson v700 or v800)?
4. General Methods questions:
  1. Can this program be adapted to analyze different sized plates?
  2. Is there a particular thickness the agar needs to be or will any depth work?
  3. How does your group actually get an estimated 100 animals onto each plate?
5. Worm Strains and Maintenance should mention which exact strains/genotypes were used and acknowledge the CGC if applicable. The “ *daf-2*” strain examined later in the manuscript has no genotype
  mentioned that I can find.
6. You mention in the results section (pdf page 3) the different measurements that are available in the end product after analysis: circumference, area and circularity. None of these measurements are listed in Figure 2 – do these data appear in a different output spreadsheet? Also, in Figures 5 and 6, you mention using perimeter to calculate the length of worms. Is perimeter another measurement that is included in the automated end product after analysis? If so, please add that item to the list in the results (given that you use it in the data presented in the Figures). If not, please provide more details on how perimeter was calculated from the automated end product dataset.
7. Potential typo – page 4, under “Automated wormscan can be used to measure growth rate and fecundity” – second mention of the *daf-2* strain is missing the “ *-2*.” Also, related to #5 above, it would be best to name the full strain name and genotype again in this section.
8. The protocol used to determine fecundity in Figure 6 should be described as a section in the methods. Doing this would likely address many of my concerns/questions below in #9.
9. I have many questions and concerns related to Figure 6:
  1. I am not surprised to see that *daf-2 *mutants have fewer viable progeny than N2, but how are there only 2 juveniles per adult in the wildtype strain? How was this data collected? Does it align with data collected manually by a trained observer with a microscope? If so, it would be useful to add that comparison to this Figure.
  2.  Did you start this experiment with ~100 adults per plate and then end up with ~300 on the N2 plates? Did you remove the parents at some point or are they on these plates as well? Please clarify the number of adults and progeny per plate analyzed by the scanner as limiting the number of animals per plate was an important limitation to this tool.
  3. Did you set the size exclusion to include all animals on these plates at once? Or did you run the Automated Wormscan plug-in two times (once for the objects with >1.5mm perimeter and then again for the objects <0.5mm perimeter)?
10. Are there limitations to using this tool in lifespan studies? Can the scanners pick up the slight movement of the head or tail of older individuals that are no longer mobile?  
11. Are there any other mutations or scenarios when you would not recommend using this tool? For example, are there specific mutations prevent the animals from responding to the scanner light?

I have read this submission. I believe that I have an appropriate level of expertise to confirm that it is of an acceptable scientific standard.

# Peer review: Referee response for version 1

**DOI:** 10.5256/f1000research.11609.r20573

**Published:** 2017-03-06

**Authors:** Nathaniel Szewczyk

**Affiliations:** 1 MRC‐ARUK Centre of Excellence for Musculoskeletal Ageing Research, University of Nottingham, Nottingham, UK

There is little doubt that some *C. elegans* researchers will find the software provided by the authors to be a valuable addition in automating analysis of *C. elegans* grown on standard *C. elegans* laboratory medium. The authors provide good validation data of the principal elements of the analysis for counting and assessing non-moving worms following toxic insult in Figures 3 and 4 which should give others confidence in the ability to employ this software.

 However, I found the narrative which I viewed as largely a “sales pitch” for the software was both unneeded for this journal and distracting. Similarly, I was quite disappointed at the lack details of the software, what data is captured, how one might modify the software in obvious ways of interest to *C. elegans* researchers, and what values returned from the software might represent with regards to *C. elegans* biology. While my specific comments below may seem extensive, they are largely and broadly summarized in the preceding two sentences (e.g. make the introduction and discussion more balanced and make the methods/results/case use more descriptive/detailed).

 Specific points that should be addressed in a revision (listed more or less in the order that they came up reading the manuscript in the presented order):

1. This manuscript does not appear to comply with the software tool article template. Specifically, the Methods section is lacking both the implementation and operation sections. Additionally, the authors have opted for a Results section but mostly describe non-novel data and therefore, perhaps should have used a Use Cases section.
2. The Introduction should make some mention of other “digitisation and computational analysis of *Caenorhabditis elegans.*” It is certainly not the case that only the WormScan and The Lifespan Machine exist. Early efforts developed a “wormsorter”, I believe in the 1970s, which has now been commercialized as the COPAS ^1^ and is fairly standard in many *C. elegans* labs. A quick google search returned loads of other efforts at automation a few examples: drug discovery ^2^, gene expression ^3^, and locomotion ^4^. There are also an entire host of micro-fluidics devices that are continually being published and some reference to this is also probably warranted.
3. “ *C. elegans* is the premier model organism for ageing and toxicological research.” Needs to be changed, while I might agree with “is a premier model organism for…” it is certainly not the case that the toxicology field agrees with this statement as explained in a recent review on the worms’ role in toxicity testing ^5^. NB. This review also explains, briefly, the current state of play for large scale toxicity screening and explains why axenic medium is preferable for toxicology screening.
4. Related to point (3), If you want to make argument about the utility of this analysis for toxicology, better references for the use of *C. elegans *as a model system for toxicology should be provided, especially as your paper doesn’t really go through toxicology endpoints in much detail. The above review is one example but there are other older excellent reviews as well.
5. Related to point (2), if you want to talk about the “recent surge” of interest in automated and high throughput experimentation and analysis on *C. elegans* you really need to cite other examples, particularly microfluidics which, frankly, is where the surge is.
6. Given that your analysis relies on worm movement you really need to be explicit about the limitation of using this device for ageing studies. Specifically, worms are immobile prior to death and this can last several days. Thus, your device will generate falsely truncated survival curves in lifespan assays.
7. Given that I had a new post-doc count roughly 150,000 worms in 75 min last week I don’t find the number of animals that can be processed with this system vs. a manual system at all convincing. If you want to argue about throughput I would stick to the number of conditions that can be analysed as being where the saving is (we only looked at 5 conditions in that 75 min).
8. “Cheng *et al.*, 2006 ^7^” should be “2003”
9. A better description of what the plugins and macros “do” and “how they do it” should be included, probably in an implementation section in methods.
10. Related to point (9) there needs to be enough detail such that someone who wants to modify the software can easily do so. For example, what if someone wants to image different size petri dishes or even 24 well tissues culture?
11. Related to point (9) there needs to be more explanation of what the size parameters are likely to represent for example what likely corresponds to an L1 (can you image an L1?), and L2 (can you image an L2?), an L3, and L4, and adult, etc.
12. Related to point (9) and (11), what are circumference, area, and circularity indicative of? What do different values likely mean, what are the strengths and limitation of each measurement with regards to individual worms?
13. Check your worm count in Figure 1, the legend says approximately 100 early L4 stage worms but a quick manual count (prompted by a quick visual estimate of more than 100) suggests closer to 200. If this is closer to 200 and you are making statements about 100 worms being optimal for maximal reliability either provide a better example image or revise your statements to reflect 200 (in which case additional details of how you get the approximate number that you put down should be provided, for example are you counting worms, visually estimating, automatically putting down counted worms (as the COPAS could do)).
14. Please provide details of what parameters were used to generate the worm count in Figure 3. Are these likely to be L1, L2, L3, L4, Adult, some combination? And did visual conformation match the expectation for animal stage?
15. Similar to (14) please provide details of what parameters were used to generate the survival curve and what stage these are likely to represent and if visual conformation matched expectations. (As written your paper suggests you dosed L4 animals and they remained L4 animals but this does not match your lab’s past work so I suspect this is a lack of clarity in this paper).
16. Provide details for what strain of *daf-2* was used and temperature of cultivation.
17. Similar to (14) please provide details of what parameters were used to generate this length data and how this compares with life stage and visual confirmation. In both instances the estimated lengths seem too short for the populations to be entirely adults, this should be reflected in the title (perhaps Size after 48 hr growth from L1 is a better title?). NB. You refer to perimeter in the figure legend and results section but perimeter is not a parameter that is otherwise mentioned in the description of the software.
18. Figure 5 does not provide a growth rate as a single data point is provided. I suggest simply removing “rate” from the title may fix this problem.
19. The well-established size difference of *daf-2* strains is for width (for example Depuydt et al. ^6^) yet you are claiming your length data reflects this well established difference. Please provide a reference for how your length data match past published literature for *daf-2* length at a comparable growth point.
20. In the results you state “adults being well over 1.5mm in perimeter and juveniles being under 0.5mm.” This does not seem consistent with past published literature where L4 and young adult animals are similar in length with juveniles being in a somewhat linear distribution of smaller sizes down to the size of a newly hatched L1 and adults being in a somewhat linear distribution of larger sizes up until mid-late adulthood. Please clarify/correct this statement. N.B. this relates to point (14) and the related points all of which request more clarity of what is being measured and how it precisely relates to visual inspection.
21. Figure 6, Fecundity is typically number of eggs produced. I suggest changing the title to progeny produced or viable progeny produced in two(?) days of egg laying. The figures seem low as your wild-type data are suggesting 2 viable progeny for each adult where >200 are expected. To be honest, from the description you provide it sounds like you have measured a decline in juveniles that results from both a developmental delay in *daf-2* and decreased fecundity. Thus, you really need to be clearer about what you are really detecting and if you want to make clear claims about fecundity you should run a more appropriately controlled example.
22. If you are going to make claims about the Wormscan improving workflow etc (for example “greatly improves the speed at which such experiments can be performed”, you should discuss what other bottlenecks will occur. For example, in your set up how many plates can you put in a desicator at once? There is a reason, afterall, that high throughput systems go, largely, to liquid culturing. Similarly, do plates need to be poured at a specific depth for optimal use in the scanner, does this add time?
23. I would disagree that most *C. elegans* assays are done on NGM plates, these days molecular endpoint and/or sub-cellular endpoint are much more common than movement (perhaps excluding toxicology and/or ageing). You might revise the sentence.
24. It is certainly not true that human counting is precluded in conditions that induce aggregation. One simple disaggregates the worms to count them. This statement should be removed from the discussion.
25. Since it is clear that mobility defects impair use of the system some guidance as to how little mobility is required would be quite helpful in the discussion as would be discussion of if this can be overcome by changing the scan parameters.

I have read this submission. I believe that I have an appropriate level of expertise to confirm that it is of an acceptable scientific standard, however I have significant reservations, as outlined above.

## References

[1] PulakR: Techniques for analysis, sorting, and dispensing of C. elegans on the COPAS flow-sorting system. *Methods Mol Biol*.2006;351: 10.1385/1-59745-151-7:275 275-86 10.1385/1-59745-151-7:275 16988441

[2] O'ReillyLLukeCPerlmutterDSilvermanGPakS: C. elegans in high-throughput drug discovery. *Advanced Drug Delivery Reviews*.2014;69-70: 10.1016/j.addr.2013.12.001 247-253 10.1016/j.addr.2013.12.001 PMC401971924333896

[3] MurrayJBaoZBoyleTBoeckMMericleBNicholasTZhaoZSandelMWaterstonR: Automated analysis of embryonic gene expression with cellular resolution in C. elegans. *Nature Methods*.2008;5(8) : 10.1038/nmeth.1228 703-709 10.1038/nmeth.1228 PMC255370318587405

[4] CroninCJMendelJEMukhtarSKimYMStirblRCBruckJSternbergPW: An automated system for measuring parameters of nematode sinusoidal movement. *BMC Genet*.2005;6: 10.1186/1471-2156-6-5 5 10.1186/1471-2156-6-5 15698479PMC549551

[5] HuntPR: The C. elegans model in toxicity testing. *J Appl Toxicol*.2017;37(1) : 10.1002/jat.3357 50-59 10.1002/jat.3357 27443595PMC5132335

[6] DepuydtGXieFPetyukVAShanmugamNSmoldersADhondtIBrewerHMCampDGSmithRDBraeckmanBP: Reduced insulin/insulin-like growth factor-1 signaling and dietary restriction inhibit translation but preserve muscle mass in Caenorhabditis elegans. *Mol Cell Proteomics*.2013;12(12) : 10.1074/mcp.M113.027383 3624-39 10.1074/mcp.M113.027383 24002365PMC3861712

# Peer review: Referee response for version 1

**DOI:** 10.5256/f1000research.11609.r20569

**Published:** 2017-03-06

**Authors:** Elena M. Vayndorf

**Affiliations:** 1 Institute of Arctic Biology, University of Alaska Fairbanks, Fairbanks, AK, USA

The authors present a system, including easy-to-acquire equipment, software and instructions, for counting live, moving and stimulus responsive *C. elegans*. The authors also demonstrate that human counting vs. Wormscan system counting of animal survival produces similar results that are not significantly different. In addition, the Wormscan system is able to count worm size and live progeny number and these numbers are similar to human scoring. 

 The Wormscan system can be a useful tool for toxicological studies and teaching laboratory experiments. Overall, the authors have outlined methods for setting up the system, addressed its limitations and performed a set of proof of concept experiments that demonstrate the system’s novelty and utility. The Wormscan will make a useful teaching tool for high school and college laboratories or any laboratory looking to setup a simple, high-throughput survival *in vivo* assay. 

 I have the following questions/suggestions for the authors to consider for revision (in no particular order): 

1. In addition to the provided documentation, the authors should consider making a short video tutorial that guides the user step by step in data acquisition and analysis. What may seem «easy» or obvious, can sometimes be less so, especially when setting up the system for the first time.
2. Fig 3 - please clarify the age of the animals and add to figure legend.
3. Fig 4 - over what time period were these data collected over? Add to figure legend.
4. Figure 6 - how many adults were used for the progeny assay?
5. What happens at the very end of life/survival when the animals do not move at all but are still alive? How can the Wormscan system distinguish between living vs. dead animals? Please address this tail end of the curve. Or is this point moot because you are always comparing to the moving control group? If so, please clearly state this in your write-up. Also, in this regard, it would be interesting to determine if the Wormscan can pick up whether an animal is dead even when it is not moving on a petri dish by scanning over a longer period of time. 
6. In regard to point in #5 above, beyond toxicological studies, do you envision this system for use in aging lifespan studies? Please address in the discussion.
7. Perhaps I missed this - if the scan takes 10 minutes, are the animals exposed to a bright light for the entire duration of the time? Why does it take so long to make a single scan? Is it due to the high resolution necessary for image acquisition? In this regard, I am glad to see that overheating was not a problem, but if the time of the scan and overall stress level could be decreased, perhaps this system could be expanded to other types of experiments e.g. motility scoring? This could be an avenue to explore in the future, and perhaps this point could be addressed in the discussion?  
8. Is it possible to make this work with a Mac? Or does that depend on scanner compatibility? As it is clear that you have done a lot of testing of the scanner component of the Wormscan system, perhaps in the discussion, you could address the minimum scanner and PC requirements that are needed to set this up with other scanners beyond those that work on the Windows system? $500 may not seem like a lot for a scanner, but it may still be prohibitive for some researchers and teachers, so a thorough understanding of the requirements of the most expensive piece of the system, the scanner, would be most useful.  
9. «The required software for Automated WormScan can be downloaded as an easy to install complete software package available at doi, 10.5256/f1000research.10767.d152697 19 I was not able to access this link (it was circular for me and took me back the main page, I tried it from multiple sources).
10. Please clarify in figure legends and/or methods how many times experiments were repeated and how many biological replicates were completed. 
11. Consider expanding the first paragraph of or supplying addition citation to your «Introduction» by doing a more thorough review of the technologies currently available for high-throughput image acquisition and phenotyping of C. elegans. For instance, see this review by Kinser and Pincus 2016: http://www.sciencedirect.com/science/article/pii/S104474311630046X

I have read this submission. I believe that I have an appropriate level of expertise to confirm that it is of an acceptable scientific standard.
